# Mapping of the QTLs governing grain micronutrients and thousand kernel weight in wheat (*Triticum aestivum* L.) using high density SNP markers

**DOI:** 10.3389/fnut.2023.1105207

**Published:** 2023-02-10

**Authors:** Karthik Kumar Manjunath, Hari Krishna, Narayana Bhat Devate, V. P. Sunilkumar, Divya Chauhan, Shweta Singh, C. N. Mishra, J. B. Singh, Nivedita Sinha, Neelu Jain, Gyanendra Pratap Singh, Pradeep Kumar Singh

**Affiliations:** ^1^Division of Genetics, ICAR-Indian Agricultural Research Institute, New Delhi, India; ^2^ICAR-Indian Institute of Wheat and Barley Research, Karnal, India; ^3^Regional Station, ICAR-Indian Agricultural Research Institute, Indore, India; ^4^ICAR-National Bureau of Plant Genetic Resources, New Delhi, India

**Keywords:** wheat, drought, heat, combined stress, QTL, RILs

## Abstract

Biofortification is gaining importance globally to improve human nutrition through enhancing the micronutrient content, such as vitamin A, iron, and zinc, in staple food crops. The present study aims to identify the chromosomal regions governing the grain iron concentration (GFeC), grain zinc concentration (GZnC), and thousand kernel weight (TKW) using recombinant inbred lines (RILs) in wheat, developed from a cross between HD3086 and HI1500. The experiment was conducted in four different production conditions at Delhi *viz.*, control, drought, heat, and combined heat and drought stress and at Indore under drought stress. Grain iron and zinc content increased under heat and combined stress conditions, while thousand kernel weight decreased. Medium to high heritability with a moderate correlation between grain iron and zinc was observed. Out of 4,106 polymorphic markers between the parents, 3,407 SNP markers were used for linkage map construction which spanned over a length of 14791.18 cm. QTL analysis identified a total of 32 chromosomal regions governing the traits under study, which includes 9, 11, and 12 QTLs for GFeC, GZnC, and TKW, respectively. A QTL hotspot was identified on chromosome 4B which is associated with grain iron, grain zinc, and thousand kernel weight explaining the phenotypic variance of 29.28, 10.98, and 17.53%, respectively. Similarly, common loci were identified on chromosomes 4B and 4D for grain iron, zinc, and thousand kernel weight. *In silico* analysis of these chromosomal regions identified putative candidate genes that code for proteins such as Inositol 1,3,4-trisphosphate 5/6-kinase, P-loop containing nucleoside triphosphate hydrolase, Pleckstrin homology (PH) domains, Serine-threonine/tyrosine-protein kinase and F-box-like domain superfamily proteins which play role in many important biochemical or physiological process. The identified markers linked to QTLs can be used in MAS once successfully validated.

## Introduction

Hidden hunger, often referred as micronutrient deficiency, occurs due to inadequate intake of minerals like iron, zinc, iodine, and vitamin A. The main cause of hidden hunger is the consumption of energy-rich but nutrient-poor diet. According to reports, 2 billion people suffer from micronutrient deficiency worldwide (1). The devastating effects of malnutrition are seen in underdeveloped and developing countries with low-income levels and reduced dietary diversification (2). Essential micronutrients are required in minute quantities for a healthy life, otherwise, deficiencies will lead to morbidity and mortality (3).

Among essential micronutrients, iron is the most important element needed for oxygen transportation, synthesis and repair of DNA (4). The deficiency of iron causes anemia around 32.9% of people suffer from anemia worldwide (5). The recommended average daily intake of iron is 27 mg per day for pregnant women, 18 mg for women, 9 mg for lactating mother, and 8 mg for men (6). Zinc is a trace mineral that acts as a catalyst for many enzymes, restore impaired energy metabolism, and helps to regulate gene expression (7). Zinc deficiency leads to alopecia, poor growth, and sexual dysfunction (8). It’s reported that 17.3% of the world’s population is affected by zinc deficiency (9). The average recommended intake of zinc is 8 mg for women, 11 mg for men and pregnant women, 12 mg for lactating mother (6).

Wheat is a staple food crop and the second-largest grain in both acreage and production worldwide. It’s the second largest consumed food grain and accounts for 20% of protein and calories (10). The wheat cereal-based diets fail to provide the necessary amount of minerals like iron and zinc (11). Hidden hunger is more pronounced in the areas with cereals as sole source of food supply, low dietary diversification and unavailability of biofortified varieties (12). Biofortification is the most ideal as it is cost-effective and sustainable way to improve grain micronutrient concentration (13). Therefore, attention should be given to develop biofortified varieties using breeding and molecular techniques (14).

Enrichment of grain micronutrients through traditional breeding approach is difficult because of polygenic inheritance and interaction with environment. With the advent of novel molecular techniques breeders have put a step forward in utilizing markers for assessment of genetic diversity, germplasm characterization, identification of QTLs and their utilization in practical plant breeding. Identification of loci governing GFeC and GZnC with high PVE and the markers linked to them will be very useful in molecular breeding. Several QTLs have been identified previously from the populations derived from crosses between tetraploid and hexaploid wheat varieties (15–18). Few biofortified wheat varieties released so far in India include, WB2 and HPBW 01 (high Zn and Fe), PBW1Zn (high Zn content), Pusa Tejas and Pusa Ujala (high protein content along with Zn) (19). However, only a few QTLs are identified in wheat with high phenotypic variance and are hardly being used in Indian molecular breeding for grain nutrient improvement. Hence, the present study aims to identify the novel and stable QTLs for grain zinc concentration, grain iron concentration and thousand kernel weight using mapping population derived from a cross between HD3086 and HI1500. The study was conducted across the environments viz, Delhi and Indore, in control and stress conditions (drought, heat, and combined drought and heat).

## Materials and methods

### Plant material and environment

The mapping population consists of 166 recombinant inbred lines (RILs) derived from a cross between HD3086 and HI1500. HD3086 is an Indian high-yielding hexaploid wheat variety suitable for timely sown, irrigated condition developed at IARI, New Delhi (20, 21). HI1500 is a popular variety recommended for cultivation under restricted irrigation in central zone harboring many important traits related to drought and heat tolerance (21, 22). The variety HI1500 has higher GFeC, GZnC, and TKW (44.50 mg/kg GFeC, 53.50 mg/kg GZnC, and 35.88 g TKW) in all the conditions as compared to HD3086 (38.60 mg/kg GFeC, 38.60 mg/kg GZnC, and 35.58 g TKW). The RILs population along with parents were evaluated under four conditions namely timely sown irrigation (TSIR) taken as control, timely sown restricted irrigation (TSRI), late sown irrigation (LSIR), and late sown restricted irrigation (LSRI) conditions at Delhi, and under restricted irrigation condition at Indore. Two irrigations were given in TSRI, one at germination and other at 21 days after sowing; six irrigations were provided during cropping period in irrigated condition (TSIR). Late sown trials (LSIR and LSRI) were planted in second fortnight of December to expose plants to heat stress, and under LSRI condition irrigation was withheld to expose plants to both terminal heat and drought stress. The genotypes were evaluated in an alpha-lattice design with two replications. Each genotype was sown in 3 rows of 1 m each with 22.5 cm distance between rows and 10 cm distance between plants. Uniform agronomic practises were practiced for proper establishment of crop stand. The details of the sowing conditions and locations are presented in Table 1.

**TABLE 1:** Details of the experiment locations, sowing conditions of the mapping population.

Location	Condition		Treatment
Delhi	Timely sown (first fortnight of November)	Irrigated (6 irrigations)	Control
		Restricted irrigation (2 irrigations)	Drought
	Late sown (second fortnight of December)	Irrigated (6 irrigations)	Heat
		Restricted irrigation (2 irrigation)	Combined stress
Indore	Timely sown (first fortnight of November)	Restricted irrigation (2 irrigations)	Drought

### Phenotyping for grain micronutrients and thousand kernel weight

From each plot, 20 random spikes were harvested and spikes from each plot were threshed separately. While cleaning, care was taken to prevent metal and dust contamination. The grain iron concentration (GFeC) and grain zinc concentration (GZnC) were measured using Energy Dispersive X-ray Fluorescence (ED-XRF) machine (model X-Supreme 8000 M/s Oxford Inc., USA). The thousand kernel weight (TKW) was recorded by counting 1,000 grains manually and weighted with an electronic balance.

### Phenotypic data analysis

Analysis of variance was done using PBTools v1.4 software (23). Heritability and correlations among traits were calculated using the MetaRv6.0 (Multi Environment Trial Analysis with R) software (24). From phenotypic data best linear unbiased predictors (BLUPs) were calculated for the individual conditions and combined across all production conditions and environments for further QTL mapping.

### DNA extraction and genotyping

DNA was extracted from 21 days old seedling using the CTAB method (25). Genomic DNA quality was determined using 0.8% agarose gel electrophoresis with λ DNA as the standard and quantified using nanodrop. The 35K SNP Axiom breeders’ array was used for genotyping of parents and the RILs population.

### Linkage map construction

A total of 4,106 SNP markers were polymorphic between the two parents. Among these, the redundant markers were removed by binning and markers deviating from mendelian segregation were deleted. Finally, a set of 3,407 non-redundant SNP markers spanning all over the chromosomes were used for the linkage map construction using IciMapping v4.2 software (26). Kosambi mapping function was used to calculate map distances between markers. Marker grouping was done using a rec value of 0.37 and the linear order of markers was determined. Ordering is done using function K-optimality 3-optMAP with NN initials of 10. Rippling was carried out using recombination with a window size of 5 cm. The final generated linkage map is used for QTL mapping.

### QTL analysis and identification of candidate genes

QTL mapping was performed using IciMapping 4.2 software (26) with inclusive composite interval mapping (ICIM-ADD) model (27). BLUP values were calculated in the individual environment and pooled over environments along with genotypic data used for QTL analysis. “Mean replacement” was used to address missing phenotypic data. The walking speed of 1.0 cm, with *P* = 0.001 was used in stepwise regression. The LOD score of 3.0 along with 1,000 permutations was chosen for the declaration of the QTL. Identified QTLs were named following standard nomenclature available in the catalog of wheat gene symbols (28). The candidate genes (CGs) were identified based on the positions of flanking markers of the QTL. BLAST search was done to identify putative candidate genes in the physical location of markers against IWGSC wheat (*Triticum aestivum* L.) reference genome embedded in the Ensembl Plants database.^1^

## Results

### Variability, heritability, and correlations

The parents of RIL population showed promising variability for GFeC, GZnC, and TKW. The variety HI1500 had higher GFeC, GZnC, and TKW (44.50 mg/kg GFeC, 53.50 mg/kg GZnC, and 35.88 g TKW) in all the conditions as compared to HD3086 (38.60 mg/kg GFeC, 38.60 mg/kg GZnC, and 35.58 g TKW). The parental mean difference for GFeC, GZnC and TKW was given in Table 2.

**TABLE 2:** Parental difference for the grain iron, zinc content, and thousand kernel weight.

Location	Condition	Genotype	GFeC (mg/kg)	GZnC (mg/kg)	TKW (g)
Delhi	TSIR	HD3086	38.60	38.60	35.58
		HI1500	44.50	53.50	35.88
	TSRI	HD3086	35.80	29.20	36.18
		HI1500	41.60	47.60	35.23
	LSIR	HD3086	36.80	53.90	23.40
		HI1500	40.00	64.60	32.70
	LSRI	HD3086	39.00	46.20	28.60
		HI1500	39.10	50.00	24.28
Indore	TSRI	HD3086	41.30	33.20	41.50
		HI1500	40.80	41.70	47.70

**FIGURE 1:**
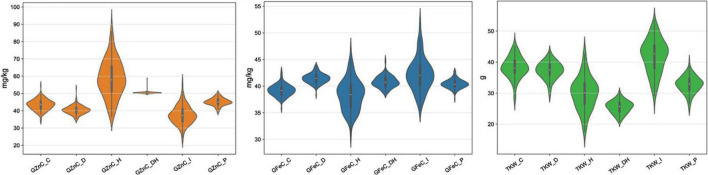
Depiction of the distribution of GFeC, GZnC, and GZnC under control, drought, heat, and combined stress conditions through violin plots.

The mean TKW of the RILs was highest in the timely sown condition compared to drought, heat and combined stress, whereas the GFeC and GZnC were low under control condition as compared to combined stress. The mean TKW in control was 38.28 g, which decreased to 37.36 g under drought, 29.99 g under heat, and 25.49 g under combined stress condition, while TKW in restricted irrigated condition at Indore increased to 41.88 g (Table 3). The GFeC under control was 39.22 mg/kg and increased to 41.51 mg/kg under drought, 40.75 mg/kg under combined stress, and 42.05 mg/kg under drought in the Indore, in contrast it was reduced to 38.41 mg/kg in heat stress. The GZnC in control was 43.31 mg/kg and increased to 59.33 mg/kg under heat and 50.44 mg/kg under combined stress, decreased to 40.46 and 37.77 mg/kg under drought condition in Delhi and Indore, respectively. Violin plots depicting GFeC, GZnC and TKW under all the studied condition are given in the Figure 1. The mean decrease in TKW and increase in GFeC and GZnC was found significant (*p*-value of 0.05) using one tailed and two tailed *z*-test (Table 3). Analysis of variance showed significant variation among the RILs for the traits GFeC, GZnC, and TKW in all the conditions *viz*., control (TSIR), drought (TSRI), heat (LSIR), and combined stress (LSRI) in Delhi and drought stress (TSRI) in Indore.

**TABLE 3:** Descriptive statistics, heritability, and percent mean increase/decrease under drought, heat, and combined stress.

Location	Condition	Trait	Grand mean	Range	LSD	CV	GCV	h^2^	Mean increase/ Reduction (%)	*z*-test
Delhi	TSIR	GFeC	39.22	35.8–42.8	3.13	5.66	2.38	0.49		
		GZnC	43.31	34.43–54.67	7.57	11.54	16.71	0.57		
		TKW	38.28	27.03–46.89	5.44	8.07	15.44	0.76		
	TSRI	GFeC	41.51	38.31–47.57	3.47	6.62	2.54	0.40	5.53	19.82**
		GZnC	40.46	34.69–53	7.95	14.95	13.97	0.43	-7.04	9.83**
		TKW	37.36	29.07–43.3	5.18	8.30	11.36	0.70	-2.47	2.68**
	LSIR	GFeC	38.41	30.62–46.9	1.21	1.59	9.99	0.98	-2.11	3.50**
		GZnC	59.33	36.3–90.76	1.91	1.63		1.00	27.01	17.65**
		TKW	29.99	16.45–44.25	3.80	6.25	29.18	0.94	-27.63	16.73**
	LSRI	GFeC	40.75	38.58–44.97	3.35	6.17	2.55	0.45	3.76	13.36**
		GZnC	50.44	49.64–58.511	8.92	48.90	12.26	0.03	14.14	28.95**
		TKW	25.49	20.71–30.22	4.88	12.60	6.97	0.58	-50.19	26.87**
Indore	TSRI	GFeC	42.05	35.52–52.31	1.58	1.93	10.00	0.97	6.73	11.65**
		GZnC	37.77	22.71–57.08	1.14	1.53	29.92	0.99	-14.65	12.09**
		TKW	41.88	27.3–53.5					8.59	7.66**

**Significant at *p* value < 0.01.

The coefficient of variation was high under drought and combined stress conditions compared to the control but heritability was found to be lower under drought and combined stress conditions for all the traits. Broad sense heritability of the traits studied was medium to high except for the GZnC which was low under combined stress condition. The range, heritability, coefficient of variation and mean decrease/increase are given in Table 3. Significant association among GFeC and GZnC under control (0.51^***^), drought (0.57^***^) and heat (0.50^***^) at Delhi location and GFeC and GZnC under drought stress (0.41^***^) at Indore (at *p*-value of 0.001) was observed. Traits viz., GFeC and GZnC did not show significant association with TKW except positive significant (0.20^**^) association between TKW and GZnC (at *p*-value of 0.01) under the drought (Table 4).

**TABLE 4:** Genotypic correlation coefficients among grain iron, zinc, and thousand kernel weight in the RIL population.

Location	Condition	Trait	Correlation		
			**GFeC**	**GZnC**	**TKW**
Delhi	TSIR	GFeC	1.00	0.51***	0.02
		GZnC		1.00	0.20**
		TKW			1.00
	TSRI	GFeC	1.00	0.57***	0.03
		GZnC		1.00	-0.02
		TKW			1.00
	LSIR	GFeC	1.00	0.50***	-0.08
		GZnC		1.00	-0.01
		TKW			1.00
	LSRI	GFeC	1.00	-0.04	0.05
		GZnC		1.00	-0.01
		TKW			1.00
Indore	TSRI	GFeC	1.00	0.41***	0.08
		GZnC		1.00	-0.02
		TKW			1.00

**Significant at *p* < 0.01; ***significant at *p* < 0.001.

### Linkage map

A total of 4,106 polymorphic markers were identified between HD3086 and HI1500, out of which 3,407 non-redundant markers uniformly distributed over all the 21 chromosomes were used for the linkage map construction. The linkage map spanned a genetic length of 14791.18 cm, ranging from 2.03 cm/marker in 1B to 9.41 cm/marker in 4D chromosome with an average marker density of 4.34 cm/marker. The highest number of markers mapped on genome B with a total of 1386 followed by A and D with 1,046 and 975 markers, respectively. Chromosome 2B had the highest number of markers 239 while chromosome 4B had only 61 markers (Table 5).

**TABLE 5:** Distribution of markers and marker density across chromosomes in the linkage map developed in RILs of HD3086/HI1500.

Chromosome	Number of SNPs	Map distance (cm)	Map density (cm/marker)
1A	89	448.96	5.04
2A	230	798.11	3.47
3A	143	801.77	5.61
4A	114	684.91	6.01
5A	189	769.34	4.07
6A	127	520.88	4.10
7A	154	737.07	4.79
1B	223	452.25	2.03
2B	239	686.47	2.87
3B	200	859.89	4.30
4B	74	530.83	7.17
5B	233	816.16	3.50
6B	213	647.2	3.04
7B	204	856.35	4.20
1D	163	659.95	4.05
2D	231	843.75	3.65
3D	151	888.83	5.89
4D	61	573.76	9.41
5D	148	875.83	5.92
6D	109	584.55	5.36
7D	112	754.32	6.74
A genome	1046	4761.04	4.55
B genome	1386	4849.15	3.50
D genome	975	5180.99	5.31
Total	3407	14791.18	4.34

### QTL mapping

A total of 32 QTLs were mapped on 17 different chromosomes for the traits GFeC, GZnC, and TKW across the environment. Out of 32, nine QTLs were mapped for GFeC, eleven for GZnC, and twelve for TKW. Chromosome 4B carried the highest number of QTLs i.e., 5, followed by chromosome 2D which carried 4 QTLs, while chromosomes 1D, 2A, 3B, 4D, 5D, 6A, 7A, and 7B carried 2 QTLs each, and the remaining chromosomes 3A, 4A, 5A, 5B, 6B, 6D, and 7D carried only one QTL. A list of QTLs identified along with flanking markers, LOD score, PVE (%), and additive effects are given in Table 6. *In silico* analysis of QTL regions identified a few important candidate genes which are having various roles in different pathways of growth and development were given in Table 7.

**TABLE 6:** QTLs identified for GFeC, GZnC, and TKW in the RIL population under control, drought, heat, and combined stress conditions.

Trait	Loc	Con	QTL name	Chr	Pos	Left marker	Right marker	LOD	PVE (%)	Add effect	Left CI	Right CI
GFeC	Delhi	TSIR	QGFeC.iari-1D.1	1D	641	AX-94648809	AX-95230812	3.86	8.28	0.409	628.5	652.5
			QGFeC.iari-2D.1	2D	281	AX-94734286	AX-95151743	5.04	5.91	0.348	276.5	284.5
			QGFeC.iari-4D.1	4D	163	AX-94638169	AX-94895991	9.78	16.90	-0.585	155.5	169.5
		TSRI	QGFeC.iari-4B	4B	335	AX-94957045	AX-95215762	14.38	29.28	-0.506	334.5	335.5
		LSRI	QGFeC.iari-5B	5B	0	AX-95104064	AX-95155233	3.63	5.57	0.310	0	17.5
			QGFeC.iari-6B	6B	41	AX-94539326	AX-94842542	3.68	5.86	0.319	40.5	42.5
			QGFeC.iari-1D.1	1D	649	AX-94648809	AX-95230812	3.55	11.48	0.445	637.5	659
	Indore	TSRI	QGFeC.iari-1D.2	1D	604	AX-94901148	AX-94545049	5.17	9.30	1.136	598.5	605.5
	Pooled		QGFeC.iari-2D.2	2D	539	AX-94412799	AX-94955614	4.98	6.32	0.236	538.5	539.5
			QGFeC.iari-4D.1	4D	168	AX-94638169	AX-94895991	12.40	17.87	-0.396	161.5	169.5
			QGFeC.iari-6D	6D	223	AX-94614625	AX-95177090	4.33	8.63	0.275	211.5	231.5
GZnC	Delhi	TSIR	QGZnC.iari-3B	3B	486	AX-94791212	AX-95256225	4.15	10.78	-0.880	480.5	494.5
			QGZnC.iari-4B.1	4B	346	AX-94461604	AX-95115431	4.70	9.19	-0.793	345.5	348.5
			QGZn.iari-7B.1	7B	269	AX-94712454	AX-95076244	7.02	14.09	-1.000	262.5	277.5
		TSRI	QGZnC.iari-3A	3A	412	AX-95125642	AX-94565216	5.36	8.08	-0.699	411.5	413.5
			QGZnC.iari-7A	7A	635	AX-94848583	AX-94488051	3.40	5.82	-0.598	629.5	641.5
			QGZnC.iari-4B.2	4B	335	AX-94957045	AX-95215762	6.72	10.98	-0.815	334.5	335.5
			QGZnC.iari-7B.2	7B	131	AX-94527140	AX-94383404	4.57	6.91	0.655	127.5	131.5
			QGZnC.iari-2D	2D	843	AX-95227268	AX-94661194	3.97	6.10	-0.610	831.5	843
			QGZnC.iari-7D	7D	326	AX-94514902	AX-94534665	6.91	10.87	-0.811	324.5	327.5
	Pooled		QGZnC.iari-5A	5A	286	AX-94635713	AX-94556600	15.06	18.29	1.285	284.5	286.5
			QGZnC.iari-5D	5D	723	AX-94724718	AX-94656442	8.15	9.73	-0.960	719.5	725.5
TKW	Delhi	TSIR	QTKW.iari-4D.1	4D	168	AX-94638169	AX-94895991	7.29	12.69	-1.333	161.5	169.5
		LSIR	QTKW.iari-4B.1	4B	335	AX-94957045	AX-95215762	6.16	17.53	-1.986	334.5	335.5
		LSRI	QTKW.iari-4A	4A	459	AX-94450390	AX-94606134	3.89	7.60	-0.563	453.5	459.5
		LSRI	QTKW.iari-6A.1	6A	215	AX-94925474	AX-94761286	4.17	7.82	0.580	214.5	215.5
		LSRI	QTKW.iari-2D	2D	586	AX-95238815	AX-94500838	4.25	8.10	0.571	582.5	588.5
	Indore	TSRI	QTKW.iari-3B	3B	463	AX-94940814	AX-94962244	3.81	10.18	1.796	462.5	463.5
	Pooled		QTKW.iari-2A.1	2A	302	AX-94505180	AX-95176936	13.90	12.53	1.180	298.5	303.5
			QTKW.iari-2A.2	2A	311	AX-94620950	AX-94422071	7.06	5.78	-0.798	307.5	312.5
			QTKW.iari-6A.2	6A	218	AX-94543425	AX-94974293	8.11	6.59	0.880	216.5	219.5
			QTKW.iari-7A	7A	36	AX-94775539	AX-94441851	5.60	6.53	-0.850	31.5	44.5
			QTKW.iari-4B.2	4B	102	AX-94991164	AX-94779755	6.61	5.32	-0.777	100.5	110.5
			QTKW.iari-4D.1	4D	168	AX-94638169	AX-94895991	15.35	13.90	-1.236	163.5	168.5
			QTKW.iari-5D	5D	507	AX-94965765	AX-94618899	3.73	4.60	-0.712	488.5	519.5

**TABLE 7:** Putative candidate genes identified in the QTL region and proteins produced.

QTL	SNP id	Chr	Transcript id	Position	Protein
QGFeC.iari-4B	AX-94957045	4B	TraesCS4B02G056800	4B: 46,615,028-46,621,497	Inositol 1,3,4-trisphosphate 5/6-kinase, ATP-grasp domain
	AX-95215762	4B	TraesCS4B02G043900	4B: 31,874,862-31,877,696	P-loop containing nucleoside triphosphate hydrolase
QGFeC.iari-1D.1	AX-94648809	1D	TraesCS1D02G240700	1D: 330,545,811-330,555,264	Regulator of chromosome condensation 1/beta-lactamase-inhibitor protein II
QGFeC.iari-2D.1	AX-94734286	2D	TraesCS2D02G044900	2D: 16,354,095-16,358,194	P-loop containing nucleoside triphosphate hydrolase
	AX-95151743	2D	TraesCS2D02G053300	2D: 16,354,095-16,358,194	Bulb-type lectin domain superfamily
QGFeC.iari-4D.1	AX-94895991	4D	TraesCS2D02G053300	2D: 20,768,191-20,770,988	Protein kinase-like domain superfamily
			TraesCS4D02G039500	4D: 16,914,929-16,924,027	Oxysterol-binding protein superfamily
			TraesCS4D02G03950	4D: 16,914,929-16,924,027	Pleckstrin homology domain
QGFeC.iari-5B	AX-95104064	5B	TraesCS5B02G491600	5B: 659,813,764-659,821,171	P-loop containing nucleoside triphosphate hydrolase
			TraesCS5B02G491600	5B: 659,813,764-659,821,171	PPM-type phosphatase domain superfamily
			TraesCS5B02G491600	5B: 659,815,819-659,821,696	Apoptotic protease-activating factors, helical domain
			TraesCS5B02G491600	5B: 659,815,819-659,821,696	Winged helix-like DNA-binding domain superfamily
			TraesCS5B02G491600	5B: 659,815,819-659,821,696	Leucine-rich repeat domain superfamily
			TraesCS5B02G494300	5B: 662,023,972-662,032,159	Protein kinase-like domain superfamily
QGFeC.iari-6B	AX-94539326	6B	TraesCS6B02G451100	6B: 710,001,532-710,007,317	Peptidase A22B, signal peptide peptidase
			TraesCS6B02G451100	6B: 710,001,532-710,007,317	Presenilin/signal peptide peptidase
	AX-94842542	6B	TraesCS6B02G445400	6B: 707,246,945-707,250,303	P-loop containing nucleoside triphosphate hydrolase
			TraesCS6B02G445400	6B: 707,246,945-707,250,303	Winged helix-like DNA-binding domain superfamily
QGFeC.iari-2D.2	AX-94412799	2D	TraesCS2D02G282900	2D: 355,685,478-355,687,949	Mediator of RNA polymerase II transcription subunit 30
	AX-94955614	2D	TraesCS2D02G228000	2D: 196,707,566-196,711,453	Helicase superfamily 1/2, ATP-binding domain
			TraesCS2D02G228000	2D: 196,707,566-196,711,453	Domain of unknown function DUF4217
			TraesCS2D02G228000	2D: 196,707,566-196,711,453	P-loop containing nucleoside triphosphate hydrolase
QGFeC.iari-4D.1	AX-94895991	4D	TraesCS4D02G039500	4D: 16,914,929-16,924,027	Oxysterol-binding protein
			TraesCS4D02G039500	4D: 16,914,929-16,924,027	Pleckstrin homology domain
QGFeC.iari-6D	AX-94614625	6D	TraesCS6D02G364100	6D: 454,124,979-454,130,612	SPX domain
			TraesCS6D02G364100	6D: 454,124,979-454,130,612	EXS, C-terminal
	AX-95177090	6D	TraesCS6D02G402700	6D: 470,990,689-471,005,705	Cell morphogenesis central region
QGFeC.iari-1D.2	AX-94901148	1D	TraesCS1D02G043700	1D: 22,883,248-22,888,807	Saf4/Yju2 protein
QGZnC.iari-3A	AX-95125642	3A	TraesCS3A02G326200	3A: 571,407,220-571,412,238	F-box-like domain superfamily
QGZnC.iari-7A	AX-94848583	7A	TraesCS7A02G489000	7A: 679,229,757-679,231,428	Aspartic peptidase domain superfamily
			TraesCS7A02G489000	7A: 679,229,757-679,231,428	Xylanase inhibitor, N-terminal
			TraesCS7A02G489100	7A: 679,362,216-679,363,352	Peptidase family A1 domain
QGZnC.iari-4B.2	AX-94957045	4B	TraesCS4B02G056800	4B: 46,615,028-46,621,497	Inositol-tetrakisphosphate 1-kinase, N-terminal
	AX-95215762	4B	TraesCS4B02G008300	4B: 5,458,308-5,460,603	Transcription factor CBF/NF-Y/archaeal histone domain
QGZnC.iari-7B.2	AX-94383404	7B	TraesCS7B02G115900	7B: 134,554,293-134,559,250	Aspartic peptidase domain superfamily
QGZnC.iari-4B.1	AX-94461604	4B	TraesCS4B02G029100	4B: 21,383,156-21,397,351	F-box domain
	AX-95115431	4B	TraesCS4B02G026300	4B: 19,745,115-19,752,157	Protein phosphatase 2A, regulatory B subunit, B56
QGZnC.iari-5A	AX-94556600	5A	TraesCS5A02G036200	5A: 33,126,001-33,130,778	NAD-dependent epimerase/dehydratase
QGZnC.iari-5D	AX-94724718	5D	TraesCS5D02G044300	5D: 43,316,281-43,321,658	P-loop containing nucleoside triphosphate hydrolase
	AX-94656442	5D	TraesCS5D02G037800	5D: 36,761,646-36,765,059	Nuclear transcription factor Y subunit A
QTKW.iari-3B	AX-94940814	3B	TraesCS3B02G295700	3B: 474,028,727-474,032,192	Alpha/beta hydrolase fold
QTKW.iari-4A	AX-94606134	4A	TraesCS4A02G154100.2	4A: 310,241,879-310,245,711	Pumilio RNA-binding repeat
QTKW.iari-6A.1	AX-94925474	6A	TraesCS6A02G303100.1	6A: 536,255,454-536,257,920	S-adenosyl-L-methionine-dependent methyltransferase
	AX-94761286	6A	TraesCS6A02G303000.1	6A: 536,251,528-536,254,709	UBL3-like, ubiquitin domain
QTKW.iari-2D	AX-94500838	2D	TraesCS2D02G389900.1	2D: 497,361,241-497,364,738	Pentatricopeptide repeat
QTKW.iari-2A.1	AX-94505180	2A	TraesCS2A02G503900.1	2A: 733,087,802-733,091,235	P-loop containing nucleoside triphosphate hydrolase
QTKW.iari-6A.2	AX-94543425	6A	TraesCS6A02G296500	6A: 530,088,835-530,090,387	EF-hand domain pair
	AX-94974293	6A	TraesCS6A02G078800.1	6A: 48,552,372-48,556,703	Peroxisome membrane protein, Pex16
QTKW.iari-7A	AX-94441851	7A	TraesCS7A02G003600.1	7A: 1,848,902-1,852,313	Homogentisate 1,2-dioxygenase
QTKW.iari-4B.2	AX-94991164	4B	TraesCS4B02G202400.1	4B: 433,332,798-433,336,642	Ankyrin repeat-containing domain superfamily

### QTL mapping for GFeC

QTL analysis identified nine QTLs for GFeC with the LOD score ranging from 3.54 to 14.37. The number of QTLs identified in different treatments *viz*., control, drought, combined stress and pooled BLUP of all the environments were 3, 2, 3, and 3, respectively. Three major QTLs *QGFeC.iari-4D.1, QGFeC.iari-4B*, and *QGFeC.iari-1D.1* explained greater than 10 percent phenotypic variance: (i) The QTL *QGFeC.iari-4B* explained the highest phenotypic variance of 29.28% flanked by markers AX-94957045 and AX-95215762 with a 1 cm confidence interval. (ii) The QTL *QGFeC.iari-4D.1* was identified in control and pooled BLUP data and found to be stable explaining the phenotypic variance of 16.90 and 17.87%, respectively. The region is associated with two candidate genes TraesCS4D02G03950 and TraesCS4D02G03950 which code for Oxysterol-binding protein and Pleckstrin homology domain, respectively. (iii) QTL *QGFeC.iari-1D.1* was identified between markers AX-94648809 and AX-95230812 with the phenotypic variance of 11.47 and 8.28% in combined stress and control conditions, respectively. In addition to the major QTLs, six minor QTLs *QGFeC.iari-2D.1, QGFeC.iari-5B, QGFeC.iari-6B, QGFeC.iari-1D.2, QGFeC.iari-2D.2*, and *QGFeC.iari-6D* were also identified with a phenotypic variance of 5.91, 5.57, 5.86, 9.30, 6.32, and 8.63%, respectively. The chromosome regions of these QTLs were found to harbor important candidate genes encoding proteins for Bulb-type lectin domain superfamily, Winged helix-like DNA-binding domain superfamily, Protein kinase-like domain superfamily, Apoptotic protease-activating factors, Presenilin/signal peptide peptidase, Protein kinase-like domain superfamily, Peptidase A22B, signal peptide peptidase, and Leucine-rich repeat domain superfamily.

### QTL mapping for GZnC

A total of 11 QTLs were identified for GZnC with the LOD value ranging from 3.40 to 15.06 out of which *QGZnC.iari-3B, QGZn.iari-7B.1, QGZnC.iari-4B.2, QGZnC.iari-7D*, and *QGZnC.iari-5A* were the major QTLs explaining phenotypic variance greater than 10 percent. The QTL *QGZn.iari-3B* flanked with markers AX-94791212 and AX-95256225, explained 10.78% phenotypic variance. This region contains a putative candidate gene TraesCS3A02G326200 that codes for F-box-like domain superfamily proteins. The QTL *QGZnC.iari-4B.2* explained a phenotypic variance of 10.98% and the region contained candidate genes TraesCS4B02G056800 and TraesCS4B02G008300 encoding Inositol-tetrakisphosphate 1-kinase, N-terminal and Transcription factor CBF/NF-Y/archaeal histone domain, respectively. The QTL *QGZn.iari-7D* contributed 10.87% to the total phenotypic variance and had a candidate gene TraesCS7B02G115900 encoding protein belonging to the Aspartic peptidase domain superfamily. QTL *QGZn.iari-5A* explained 18.29% phenotypic variance and a candidate gene TraesCS5A02G036200 coding for protein NAD-dependent epimerase/dehydratase. The QTL *QGZn.iari-7B.1* explained a phenotypic variance of 14.09%. Six minor QTLs *viz*., *QGZnC.iari-4B.1, QGZnC.iari-3A, QGZnC.iari-7A, QGZnC.iari-7B.2, QGZnC.iari-2D*, and *QGZnC.iari-5D* were identified with phenotypic variance of 9.19, 8.08, 5.82, 6.91, 6.10, and 9.73 percent, respectively. The chromosomal regions of these QTLs harbors candidate genes that codes for Aspartic peptidase domain superfamily, P-loop containing nucleoside triphosphate hydrolase, Xylanase inhibitor, N-terminal Peptidase family A1 domain, and nuclear transcription factor Y subunit A.

### QTL mapping for TKW

A total of twelve QTLs was identified on nine different chromosomes ranging from 3.81 to 15.35 LOD value; chromosomes 2A, 4B, and 6A carried two QTLs each. The *QTKW.iari-4D.1, QTKW.iari-4B.1, QTKW.iari-3B*, and *QTKW.iari-2A.1* were the major QTLs with a phenotypic variance of 12.69, 17.53, 10.18, and 12.53 percent, respectively. The genomic region of the *QTKW.iari-3B* contains important candidate gene TraesCS3B02G295700 coding Alpha/Beta hydrolase fold protein. One stable QTL *QTKW.iari-4D.1* was identified on in control and pooled data that explained a phenotypic variance of 12.69 and 13.90%, respectively. The QTL region was found to have putative candidate gene TraesCS4D02G039500 which codes for Oxysterol-binding protein and Pleckstrin homology domain. *QTKW.iari-4A, QTKW.iari-6A.1, QTKW.iari-2D, QTKW.iari-2A.2, QTKW.iari-6A.2, QTKW.iari-7A, QTKW.iari-4B.2*, and *QTKW.iari-5D* are the minor QTLs identified with PVE of 7.60, 7.82, 8.10, 5.78, 6.59, 6.53, 5.32, and 4.60%, respectively. The chromosomal regions of the QTLs codes proteins/enzymes such as Pumilio RNA-binding repeat, S-adenosyl-L-methionine-dependent methyltransferase, UBL3-like ubiquitin domain, EF-hand domain pair, Peroxisome membrane protein (Pex16), Homogentisate 1,2-dioxygenase, and Ankyrin repeat-containing domain superfamily.

## Discussion

Micronutrient elements such as grain iron, grain zinc, are governed by many genes whose expression is influenced by the external environment. In the present study, to identify QTLs expressed in different stress conditions the experiment was carried out in drought, heat, and combined stress conditions taking timely sown environment as control. The treatment-wise highest mean for GFeC 41.51 mg/kg, GZnC 59.33 mg/kg, and TKW 41.88 g was observed in drought (Indore), heat, and drought (Indore) conditions, respectively, as observed in previous studies (29, 30). The GFeC and GZnC were increased in late sown conditions but the TKW decreased because the high temperature during grain filling caused forced maturity, reduced photosynthetic assimilation and starch content leading to the development of undernourished shriveled seeds (31). Although the grain micronutrients concentration was highest in late sown condition, the total micronutrient yield per area was highest in timely sown condition as the grain yield was highest in timely sown condition similar kind of results were also observed in a study by Velu et al. (30). GCV for GFeC ranged from 2.33 to 10.00, GCV for GZnC ranged from 12.26 to 29.92 and GCV for TKW ranged from 7.18 to 29.18 in different treatment conditions. The lower the value of GCV, the greater the influence of the environment on the expression of the particular trait whereas higher value of GCV, indicates the variation in the population mainly attributes to the genetic makeup of the individual. The CV for GFeC was ranged from 5.66 to 6.62, GZnC ranged from 1.63 to 48.90, TKW ranged from 6.97 to 15.44, similar findings were observed in previous studies by 29, 32, 33. A wide variation was observed for GFeC, GZnC, and TKW (Table 3) (17, 34, 35). Heritability is important selection parameter that aids plant breeders in determining the characters for which selection is to be performed (36). The heritability of the traits studied was found to be medium to high indicating a predominance of genotypic variance which can be exploited by selection in crop improvement, (17, 37). GFeC is significantly correlated with GZnC in all the conditions, indicating they are positively associated with each other and the selection for improvement of one character will simultaneously bring improvement in another. The results were similar to previous studies by 38, 39.

**FIGURE 2:**
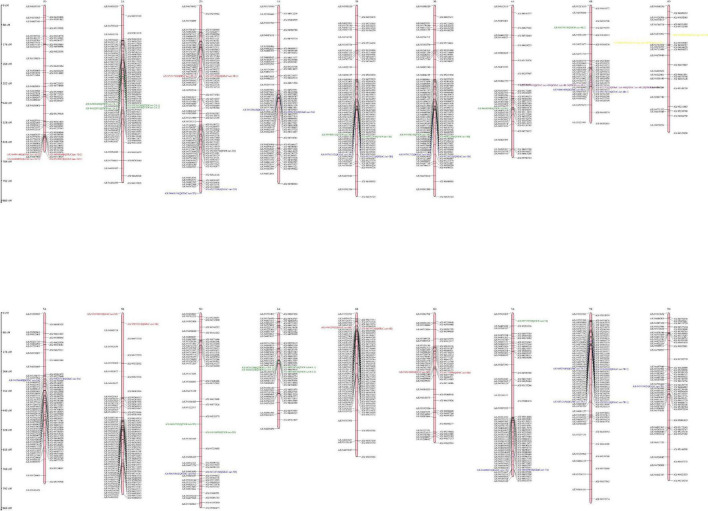
Genetic linkage map and QTL positions identified on A, B, and D genomes of RILs derived from the cross HD3086/HI1500. Red color indicates QTLs for GFeC; blue color indicates QTLs for GZnC; green color indicates QTLs for TKW; purple color indicates QTL governing GFe, GZn and TKW; yellow color indicates QTL governing both GFe and TKW.

In the present study a total of 32 QTLs were identified out of which 9, 11, and 12 for GFeC, GZnC, and TKW, respectively (Figure 2). Most of the QTLs for GZnC and TKW were derived from parent HI1500 while QTLs for GFeC were derived from parent HD3086. Interestingly, one pleiotropic QTL *QGFe.iari-4B/QGZnC.iari-4B.2/QTKW.iari-4B.1* has been identified at position 335 cm which explained the phenotypic variance of 29.28% for GFeC, 10.98% for GZnC, and 17.53% for TKW. Two Stable QTLs *QGFeC.iari-1D.1* and *QTL QGFeC.iari-4D.1* were identified for GFeC, and one for TKW *QTKW.iari-4D.1* and. were also reported in earlier studies (40–42). QTLs for grain iron content are mapped most on the D genome while B genome carried the highest number of QTLs for grain zinc content and A genome carried highest number of QTLs for TKW.

The stable QTLs for GFeC was identified on chromosome 1D and 4D as also reported by previous workers on 4D (32, 37), 1D (18). The putative candidate gene TraesCS4D02G03950 in the QTL *QGFeC.iari-4D.1* region coded Pleckstrin homology (PH) domains; these are protein modules made up of 100–120 amino acids and has the potential to bind phosphoinositides (43). Phosphoinositides are known to regulate intracellular membrane trafficking by providing intrinsic membrane signals (44) and also help in regulation of ion channel function (45). Another stable QTL *QGFeC.iari-1D.1* harbors a putative candidate gene TraesCS1D02G240700 which codes for Regulator of chromosome condensation 1/beta-lactamase-inhibitor protein II. Three major *QGFeC.iari-4D.1, QGFeC.iari-4B*, and *QGFeC.iari-1D.1* for GFeC were also reported by previous workers on 4D (32, 37), 1D (18), and 4B (37) in different mapping populations. *In silico* analysis of chromosomal regions harboring QTL *QGFeC.iari-4B* found a putative candidate gene TraesCS1D02G240700 near right marker AX-95215762 which codes for P-loop containing nucleoside triphosphate hydrolase, and belong to the special class of metallochaperones (46). Metallochaperones are specific class of molecular chaperones that mediate the intracellular transport of metal ions to metalloproteins and metalloenzymes through protein-protein interactions (47). These proteins are Fe^2+^ chaperones and important multifunctional adaptors that function in the nuclear and cytosolic compartmentalization, storage and export of iron in the form of ferritin and ferroportin (48). Another putative candidate gene TraesCS4B02G056800 codes for Inositol 1,3,4-trisphosphate 5/6-kinase, it acts as a chelator of metal ions such as iron and zinc.

Five major QTLs corresponding to GZnC *viz*., *QGZnC.iari-3B, QGZn.iari-7B.1, QGZnC.iari-4B.2, QGZnC.iari-7D*, and *QGZnC.iari-5A* were identified. The similar localization of QTL for GZnC were reported in earlier studies on 3B (29, 32, 37, 49), 7B (29, 32, 37), 4B (49), 5A (18, 29, 49), and 7D (18, 29, 37) in different mapping populations. The QTL region *QGZn.iari-3B* was found to have candidate genes coding for F-box-like domain superfamily which takes part in several biological processes like flowering, photomorphogenesis, seed development, leaf senescence, the regulation of circadian rhythms, and hormone signaling (50). The QTLs for TKW were reported in earlier studies on chromosome 3B (33, 51), 4B (51–53), 4A (51), 6A (51, 54), 2D (53, 54), 2A (33, 54), and 7A (51, 54). Candidate gene TraesCS3B02G295700 was found in the QTL *QTKW.iari-3B* region coding Alpha/Beta hydrolase fold protein. This protein serves as the core structure for phytohormone and ligand receptors in the gibberellin, strigolactone, and karrikin signaling pathways in plants (55).

## Conclusion

The number of QTLs identified for GFeC, GZnC and TKW are 9, 11, and 12, respectively. One pleiotropic QTL was identified on chromosome 4B which is associated with grain iron, grain zinc concentration and thousand kernel weight. The common locus was identified for Zn content and TKW on chromosome 4B, while common locus for Fe and TKW was found on 4D. The same genes might be functioning in the accumulation of Fe, Zn, and TKW which need to be studied further in detail at molecular and biochemical levels. Genomic regions on chromosomes 1D and 4D were associated with Fe content, 2A and 6A associated with TKW, while on chromosome 4B were associated with Zn content, therefore fine mapping of the such regions may be rewarding. Further *in silico* analysis of QTL regions identified putative candidate genes which are directly or indirectly related to these traits. So, the identified QTLs can be used in practical plant breeding to develop biofortified varieties after the successful validation of these identified markers.

## Data availability statement

The original contributions presented in this study are included in the article/Supplementary material, further inquiries can be directed to the corresponding authors.

## Author contributions

PS, NJ, GS, and HK conceptualized the investigation and edited the manuscript. KM conducted the investigation and prepared the draft of the manuscript. KM, CM, JS, and ND generated the phenotypic data. KM, HK, VS, SS, and DC contributed in the generation of genotyping data. KM and HK did the statistical and QTL analysis. All authors contributed to the article and approved the submitted version.

## Footnotes

1
https://plants.ensembl.org/index.html
